# Correction: Quantum spin models for numerosity perception

**DOI:** 10.1371/journal.pone.0331150

**Published:** 2025-08-26

**Authors:** Jorge Yago Malo, Guido Marco Cicchini, Maria Concetta Morrone, Maria Luisa Chiofalo

The following information is missing from the Funding statement: MLC acknowledges support from the National Centre on HPC, Big Data and Quantum Computing – SPOKE 10 (Quantum Computing) and received funding from the European Union Next-GenerationEU - National Recovery and Resilience Plan (NRRP) – MISSION 4 COMPONENT 2, INVESTMENT N. 1.4 – CUP N. I53C22000690001. MLC acknowledges support from the project PRA_2022_2023_98 “IMAGINATION” and acknowledges support from the MIT-UNIPI program.
