# Supplementary material for: Quantum spin models for numerosity perception
Source: PLoS One. 2023 Apr 25;18(4):e0284610. doi: 10.1371/journal.pone.0284610 (PMC10128973; doi:10.1371/journal.pone.0284610)
Supplement: S1 Appendix — (PDF) [file pone.0284610.s001.pdf]

Supporting information

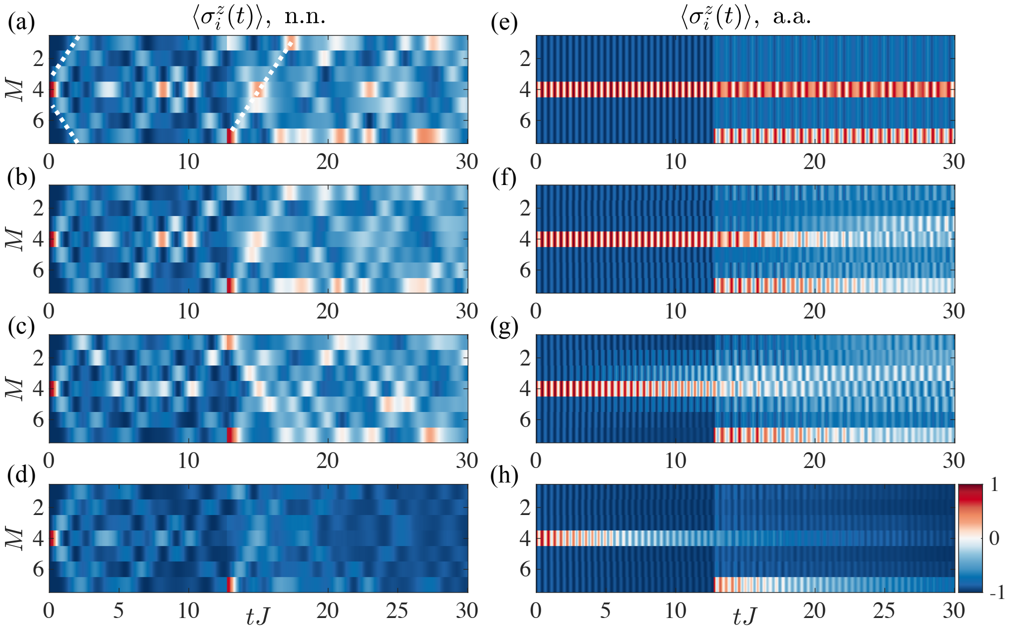

Fig 7. Adapted version of Fig 2, for the case with $N = 2$ spin flips. (a) Evolution of the local magnetisation σ_i^z for a system with $M = 7, J = 1, \Delta_0 = 0, \gamma_l = 0$ starting from a single excitation (spin up) on the middle site at $t = 0$ and a second one at time $t_1 = (11 + \sqrt{3})J$ and n.n. coupling. We use irrational numbers to prevent any symmetry-based counter-argument. The dashed lines highlight the magnetisation spreading in a light-cone manner; (b) same as (a) for $\Delta_0 = 0.1, \sigma = 1/\sqrt{2}$; (c) same as (a) for $\Delta_0 = 0.1, \sigma = 2/\sqrt{2}$; (d) same as (a) for $\gamma_l = 0.1$; (e)-(h) same as (a)-(d) for the a.a. case. In the n.n. case, both excitation cones interplay to modify the evolution profile due to interference with similar behaviour in the presence of interactions, which only shifts the specific shape of the propagation profiles but does not alter the qualitative behaviour. Inclusion of spin decay with rate $\gamma_l \neq 0$, leads to the loss of the excitations over time. The a.a. case does not exhibit light-cone spreading, but instead the excitation propagates evenly to all sites and oscillates back and forth with constant frequency. We observe how the addition of the second spin flip adds a new oscillatory frequency to the time signal, as reported in the power spectrum in Fig 3. The addition of interactions or dissipation, leading to spatial inhomogeneity, does not affect the presence of two distinct oscillation frequencies.

S1 Appendix. Discussion on Fig 8 and Fig 9. The specific cases displayed in Fig 8 highlight the robustness of the power spectrum against varying conditions, where the peaks appear with a consistent location in frequency and with a constant number of frequency modes equal to the number of stimuli. We observe that specific conditions, e.g. very small rotation angles, can reduce the amplitude of certain modes that then could lead to the decoder classifying the trajectory as the wrong numerosity. This is, in our system, the ultimate source of noise in the decoder. But these features do not limit the use of the all-to-all coupled network for the model of numerosity perception.

Moreover, in Fig 9, we present the power spectrum for the same system, for varying time windows $t_{\max} - t_1$. To avoid any correlation between data sets, each of the averages is produced with an individual set of $N_t = 100$ trajectories, each recorded for

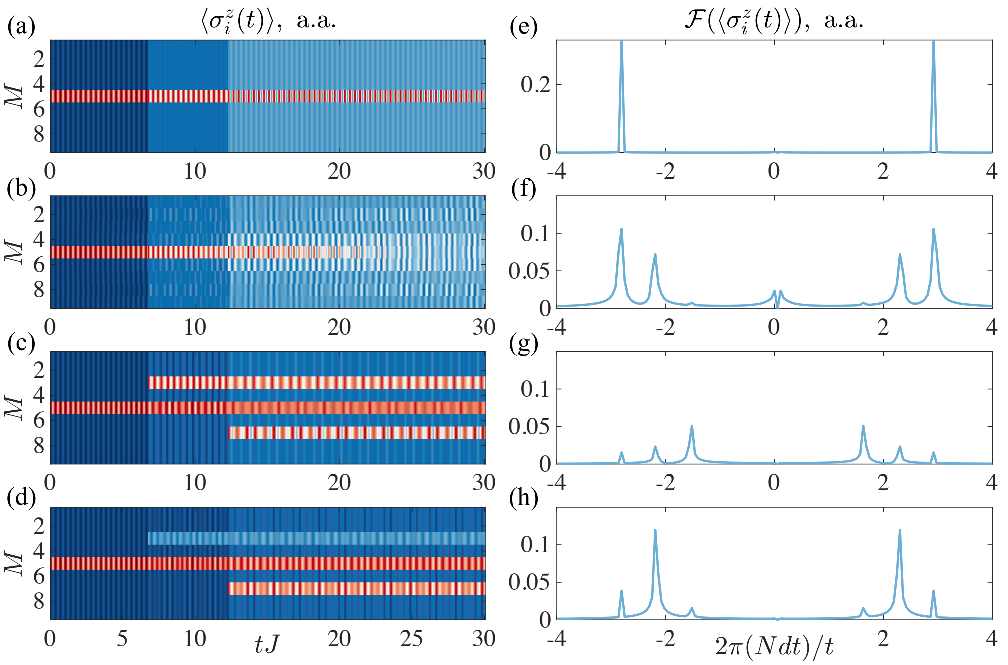

Fig 8. Spectrum dependence on rotation angle and location of stimuli. (a) Evolution of the local magnetisation σ_i^z for a system with $M = 9, J = 1, \Delta_0 = 0, \gamma_l = 0$ with spin flips in site $i = 5$ at times $t = 0$, $t_1 = (5 + \sqrt{3})J$ and $t_2 = (10 + \sqrt{5})J$; (b) same as (a) with $\Delta_0 = 0.1$; (c) same as (a) but with varying $i = 5, 3, 7$, respectively; (d) same as (c) but with a smaller rotation angle $\theta = 0.2\pi$ at t_1; (e)-(h) Amplitude spectrum associated to (a)-(d). We observe that if all spin flips occur in the same location without any spatial inhomogeneity, as in (a) and (e), the system cannot discern the number of excitations. However, as soon as the interaction, as shown in (f), modifies the spatial profile, since the energy shift depends on the number of excitations present, the system can discern the number of stimuli and produce the expected number of peaks. The rotation angle of the excitations,(c)-(d),(g)-(h), does not modify the number of peaks or their location, but only the relative amplitude.

the indicated time. The time windows were always chosen after the last stimulus presentation at time t_1. From the analysis of spectra at different system sizes, we observed that the system is producing peaks from temporal frequencies $f \sim 2\pi J$ to $f \sim 2\pi J/M$ that to be well-detected required a window larger than $\sim 2J^{-1}$ for M=9 spins (or $\sim 3J^{-1}$ for $M = 18$ as our results in Fig. 5). In the simulation we considered a minimum temporal window of $10J^{-1}$, corresponding to several cycles of the lowest temporal frequency for the largest numerosity and system size to ensure visibility.

We observe that the results depend very weakly on this time window. We do observe a small drift towards the DC component (that we eliminate in our analysis) while the time window increases. However, this only slightly reduces the relative peaks height, that in most cases remains within error bars. This result allows us to confidently perform the analysis illustrated in the main text, with time windows of the order of $t \sim 10J$.

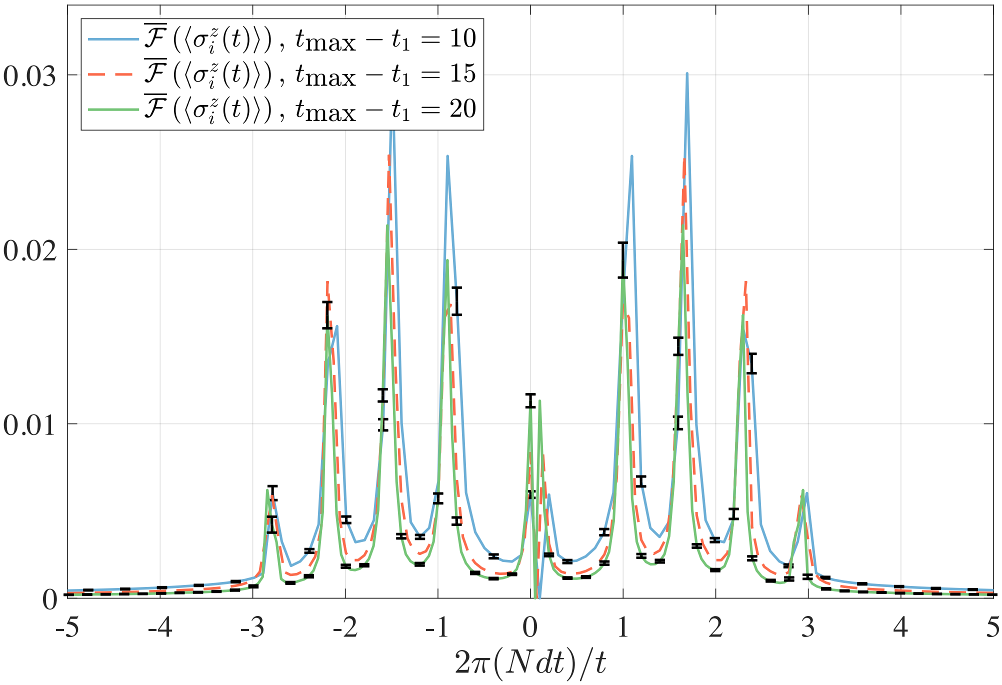

Fig 9. Spectrum dependence on recorded time window. Amplitude spectrum of the magnetisation $\langle\sigma_i^z\rangle$ as a function of the specific time window, averaged over all sites in a system with $M = 9, N = 4, J = 1, \Delta_0 = 0.1, \sigma = 1/\sqrt{2}, \gamma_l = 0$ and for varying time integration windows $t_{\max} - t_1$. The chosen parameters serve as an example and do not qualitatively change the results displayed. Each line corresponds to the average of $N_t = 100$ trajectories. We observe that the time window plays no role for the number of peaks in the spectrum, only the relative amplitude is modified. Longer time windows shift the amplitude towards the continuous (DC) component, which we eliminate in our spectrum analysis.
