# Supplementary material for: Quantum spin models for numerosity perception
Source: PLoS One. 2023 Apr 25;18(4):e0284610. doi: 10.1371/journal.pone.0284610 (PMC10128973; doi:10.1371/journal.pone.0284610)
Supplement: Attachment — Submitted filename: Response to Reviewers.pdf [file pone.0284610.s002.pdf]

We would like to thank the editor and the referee of our manuscript for the positive evaluation of our work and their valuable feedback that, as we believe, contributed to clarify the message and content of our paper. We address all of the comments from the referee individually below.

For clarity, together with this reply, we include a pdf highlighting all the differences with the original submission.

Reviewer #1: SUMMARY
In this paper, the authors introduce an original processing model based on quantum-spin dynamics that can simulate some phenomena related to numerosity perception, such as global sensitivity to numerical information and adherence to Weber's law in numerosity estimation. The proposed model can (approximately) encode the number of stimuli that it has been exposed to as peaks in its frequency spectrum, suggesting that representations of numerosity information might emerge from relatively simple processing architectures.

COMMENTS
I enjoyed reading this manuscript, which is well-written and well organized. The text is concise and successfully highlights the main aspects of interests of the research work. The results are clearly presented and discussed, and overall the findings appear aligned with the hypotheses proposed by the authors. I also appreciate the interdisciplinary nature of this contribution.
Still, I have a few major concerns that should be addressed to make the paper stronger:

1. The authors argue that current modelling literature has struggled to provide a simple architecture carrying out numerosity perception. However, several recent models have actually shown that numerosity perception could indeed emerge following unsupervised learning in generative neural networks (for a survey, see [1]). Such architectures are much simpler than the CNN-based models mentioned by the authors and are grounded on the theory of statistical mechanics: the building blocks are often energy-based networks derived from spin-glass models, whose dynamics is governed by a Hamiltonian similar to the one described by Eq. 1 in the present work (for theoretical introductions, see [2], [3]). Furthermore, a distinguishing feature of such modeling approach (that is lacking in the present proposal) is that it can explain how network plasticity (i.e., learning) could improve number acuity over time [4]. The authors should acknowledge and discuss these computational models of numerosity perception in their manuscript.

Thanks for this comment. For the sake of conciseness in our original review of the field we only reported the most recent studies based on CNN, omitting some of the pioneering work. Following the referee's suggestion we now discuss these earlier results in the introduction, as these are a pertinent reference, specially given that some of the early work introduced by Hopfield in NN also relays heavily on Hamiltonian models derived from physics. With the crucial difference that, up to our knowledge, these models when connected to neuroscience have remained in the realm of classical physics. We highlight as well some differences with the mentioned unsupervised learning modeling efforts of Zorzi and Testolin with our model. In our case, our focus is not in changing connectivity weight or do so dynamically, but we rely on the capability of symmetric small-sized networks to encode complex pattern of inputs.

2. Many scientists have recently questioned the existence of a "number sense", proposing instead that numerosity estimation is carried out by integrating non-numerical magnitudes (e.g., [5], [6]). It would be important to discuss how the proposed modeling framework could account for the influence of non-numerical cues, and possibly show that model responses are based on numerosity rather than other magnitudes embedded in the input signal. Notably, the modeling framework mentioned above has also been successfully used to simulate the influence of visual cues in numerosity perception [7], so I encourage the authors to consider this when further elaborating on such aspects.

We appreciate this comment that encourages and highlights the possibility of benchmarking and testing the robustness of our model in further scenarios in numerosity perception. While, the current project aimed at providing a proof-of-concept example of how quantum modelling — even with very few information processing units or qubits/ spins — could be applied to the perception of numerosity.

We are well-aware of the issue of non-numerical cues, like overall energy, density and area of stimulation that can bias or be used to evaluate numerosity. Indeed our model, as we describe, was analyzed both with normalized overall energy or/and including random energy excitations to contaminate the input. This was tested to guarantee that the performance is not based on overall energy (see results in Fig. 5.(e)-(f)). Some of the other possible tests are harder to perform given the fact that our model is a many-body QM network. This includes control for element size (simultaneous excitation of many nodes) or tests over constant area (that we kept constant over space and time), given that the total number of excitations is constraint by the system size and we have only considered analysis based on exact methods. With current computational resources modeling the system with a dimensionality greater than 20-25 elements, including dissipation, becomes numerically rather costly and would require the use of approximate methods. We address these issues in the discussion, citing the relevant literature.

3. The authors argue that their framework is not supervised, but the decoding procedure described at pg. 7 assumes that the library of previously learned power spectra are labeled ("associated with a given numerosity"). I think this point should be clarified or rephrased.

We recognize the lack of clarity regarding this point and understand the potential confusion.

The referee indeed is correct: the decoder employed here, is "supervised" as it learns the patterns starting from a set of labelled magnetization spectra. However the supervised decoder is not a part of the model, but has been used to asses that the QM model is capable of encoding well the numerosity. For this reason, we had to use an ideal-observer decoder that could detect small deviation and hence used a supervised classification model.

In principle, once it has been established that the QM network is changing the temporal pattern of rhythmic excitations as function of the input numerosity (not energy), many strategies (also not simulating an ideal observer) or additional computational models can be used to evaluate the encoded numerical information.

We now make clear that the section “Estimation Protocol” is an evaluation stage of the behaviour of the model and pointing to the supervised nature of the decoder and not of the model.

4. The authors should better discuss why the proposed “all-to-all” connectivity could be considered a plausible topology for carrying out numerosity perception, and whether such architecture is supported by neurobiological findings.

We thank the referee for this comment and we agree with the fact that this is a somewhat surprising feature of our model that we intend to characterise and understand in future work. We nevertheless believe that this is still plausible as in the brain, at all cortical stages and between cortical levels, there are both feedforward and feedback projections and these could propagate the information amongst all neurons of a given layer.

While we do believe that intermediate scenarios, like an all-to-all model with heterogeneous connectivity or several all-to-all subsets sparsely connected, could be successful and would also be plausible biologically. However, in this case we wanted to focus on a proof-of-principle analysis as a wide range of new ingredients were already discussed. Thus, we restrict to commenting some of these aspects in the discussion.

5. It is shown that the model dynamics is robust to manipulations of the time window. I did not fully understand whether the time window could play a role in simulating reaction times, or whether the time required for the accumulation of numerosity information is constant regardless of the chosen time window. It would be interesting to further comment on this aspect.

The time window that we consider is always post last stimulus onset and the specific interval chosen is related to the properties of the spectra that we characterize. In particular, the interval should be long enough to record at least several cycles of the lowest temporal frequency corresponding in our model to the one encoding the largest numerosity — which corresponds to half the dimensionality of the QM network. Thus, a short window inevitably will corrupt the encoding of the largest numerosity. We clarify this in the manuscript when discussing Figure 9 in the SI section and in its caption.

On the other hand, reaction times (RT) of human and animal behavior possess time components of sensory analysis, of decisional processes and of motor response. How the RT can reveal only the sensory integration time or post-diction is far from clear and depends on the specific experimental paradigm. We believe this issue is not central to our work and we have not so far explored how to potentially model it in our system.

MINOR COMMENTS
- Lines 38-39: bad phrasing
- Line 68: “has been” -> “have been”

Addressed.

References
[1] M. Zorzi and A. Testolin, “An emergentist perspective on the origin of number sense,” Philos. Trans. R. Soc. B Biol. Sci., vol. 373, no. 1740, 2018

[2] D. Ackley, G. E. Hinton, and T. J. Sejnowski, "A learning algorithm for Boltzmann machines," Cogn. Sci., vol. 9, no. 1, pp. 147–169, 1985
[3] E. Agliari, A. Barra, A. Galluzzi, D. Tantari, and F. Tavani, "A walk in the statistical mechanical formulation of neural networks," in Proceedings of the International Joint Conference on Computational Intelligence, 2014
[4] A. Testolin, W. Y. Zou, and J. L. McClelland, "Numerosity discrimination in deep neural networks: Initial competence, developmental refinement and experience statistics," Dev. Sci., vol. 23, no. 5, Sep. 2020
[5] T. Leibovich, N. Katzin, M. Harel, and A. Henik, "From 'sense of number' to 'sense of magnitude' - The role of continuous magnitudes in numerical cognition," Behav. Brain Sci., vol. 164, 2017
[/6] T. Gebuis and B. Reynvoet, "The interplay between nonsymbolic number and its continuous visual properties.," J. Exp. Psychol. Gen., vol. 141, no. 4, pp. 642–8, Nov. 2012
[7] A. Testolin, S. Dolfi, M. Rochus, and M. Zorzi, "Visual sense of number vs. sense of magnitude in humans and machines," Sci. Rep., vol. 10, no. 1, pp. 1–13, 2020
